# Recent Developments in Photoinduced Decarboxylative Acylation of α-Keto Acids

**DOI:** 10.3390/molecules29163904

**Published:** 2024-08-18

**Authors:** Shuaiqi Lu, Yilong Xiang, Jingfu Chen, Chao Shu

**Affiliations:** 1 State Key Laboratory of Green Pesticide, Engineering Research Center of Photoenergy Utilization for Pollution Control and Carbon Reduction, CCNU-uOttawa Joint Research Centre, College of Chemistry, Central China Normal University (CCNU), 152 Luoyu Road, Wuhan 430079, China; 2 China National Standard Pharmaceutical Corporation Limited, Huangshi 435002, China; 3 Key Laboratory of Functional Molecular Solids, Ministry of Education, Anhui Normal University, Wuhu 241002, China

**Keywords:** α-keto acids, photocatalysis, decarboxylative, acylation, radicals

## Abstract

Ketones are ubiquitous patterns found in various biological molecules and natural products. In recent years, a number of acylation methods have been developed based on the use of α-oxocarboxylic acids as acyl-transfer reagents, with particular emphasis on the photoinduced decarboxylative acylation of *α*-keto acids. This review focuses on the latest advancements in acylation methodologies through the decarboxylation of *α*-keto acids over the past several years, highlighting their product diversity, selectivity, and applicability. Where possible, the mechanistic rationale is presented, providing a positive outlook for the promising future of this field.

## 1. Introduction

Functionalized carbonyl compounds are important intermediates in both fundamental organic synthesis and the production of a wide range of chemical and life science products. For example, carbonyl motifs are present in thousands of natural products, bioactive molecules, pharmaceuticals, agricultural chemicals, and functional materials [1,2,3,4,5]. Therefore, the synthesis of carbonyl compounds continues to be an intriguing and relevant challenge in the context of advancing synthetic methodologies. Carboxylic acids, known as easily available feedstocks, have been extensively utilized for introducing carbonyl groups into organic molecules. Specifically, α-keto acids are frequently used as acylating agents to introduce a carbonyl group into organic molecules through decarboxylative reactions.

In recent years, significant advancements have been made in the photoinduced decarboxylation reactions of α-keto acids for the synthesis of keto-containing frameworks, benefiting greatly from the substantial advantages of visible light catalysis, including mild conditions and environmentally friendly, sustainable, and simple operations. Despite this, over the past decades, only a handful of reviews on transformations of α-keto acids have been reported [6,7,8,9,10]. However, there is no special review that summarizes the latest progress for the photoinduced decarboxylative acylation of α-keto acids over the past several years. Thus, the purpose of this review is to provide an almost ten-year survey of the representative development methodologies for the photoinduced decarboxylative acylation of α-keto acids by highlighting their product diversity, selectivity, and applicability. The mechanistic rationale is presented where possible, which has the potential to contribute to drug synthesis or late-stage functionalization. We hope that this mini-review will provide a comprehensive summary of this special topic and promote the wider development and application of α-keto acids in the future.

## 2. Decarboxylative Acyclic Acylation of α-Keto Acids with Alkenes

### 2.1. Hydroacylation of Alkenes

**Scheme 1:**
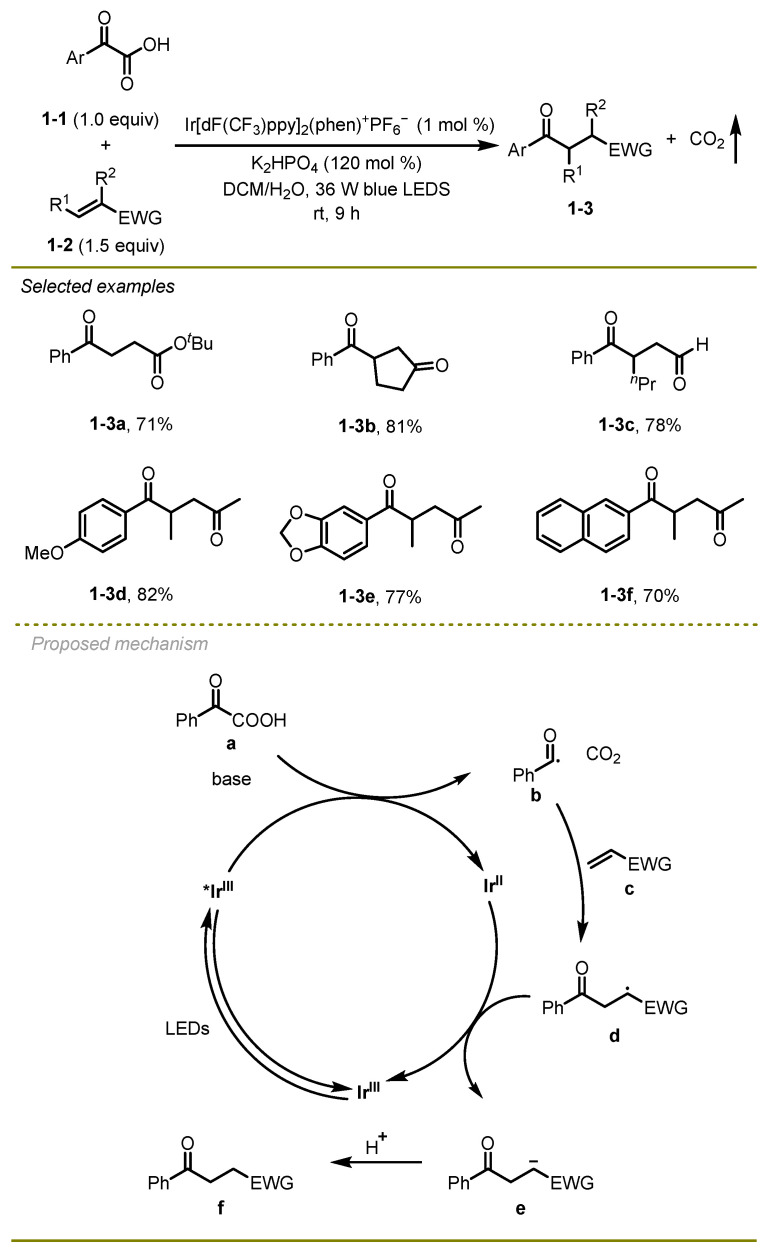
Photocatalyst iridium-catalyzed acyl decarboxylative Michael addition.

In 2015, Fu and Shang’s group published a study on a photocatalyst iridium-catalyzed acyl decarboxylative Michael addition. The study focused on the reaction of acyl-substituted α-keto acids with Michael acceptors, including unsaturated esters, sulfones, and aldehydes (Scheme 1) [11]. A wide range of α, β-unsaturated compounds, such as alkyl acrylate (**1-2a**) and 2-hexenal (**1-2c**), were well suited to this reaction, delivering high yields of the desired products. α-keto acids with either electron-donating or electron-withdrawing (**1-2d**, **f**) substituents were also tolerant to this reaction.

Mechanistically, the excited state of Ir(III) photocatalyst would initially undergo reductive quenching process to generate benzoyl radical **b**. Then, the addition of the benzoyl radical **b** onto electron-poor alkenes **c** closely followed a reduction of the resulting acyl radical species **d** with the aid of Ir(II) species, resulting in the formation of an anion intermediate **e**, which could finally trap a proton to afford the corresponding 1, 4-addition product **f**.

**Scheme 2:**
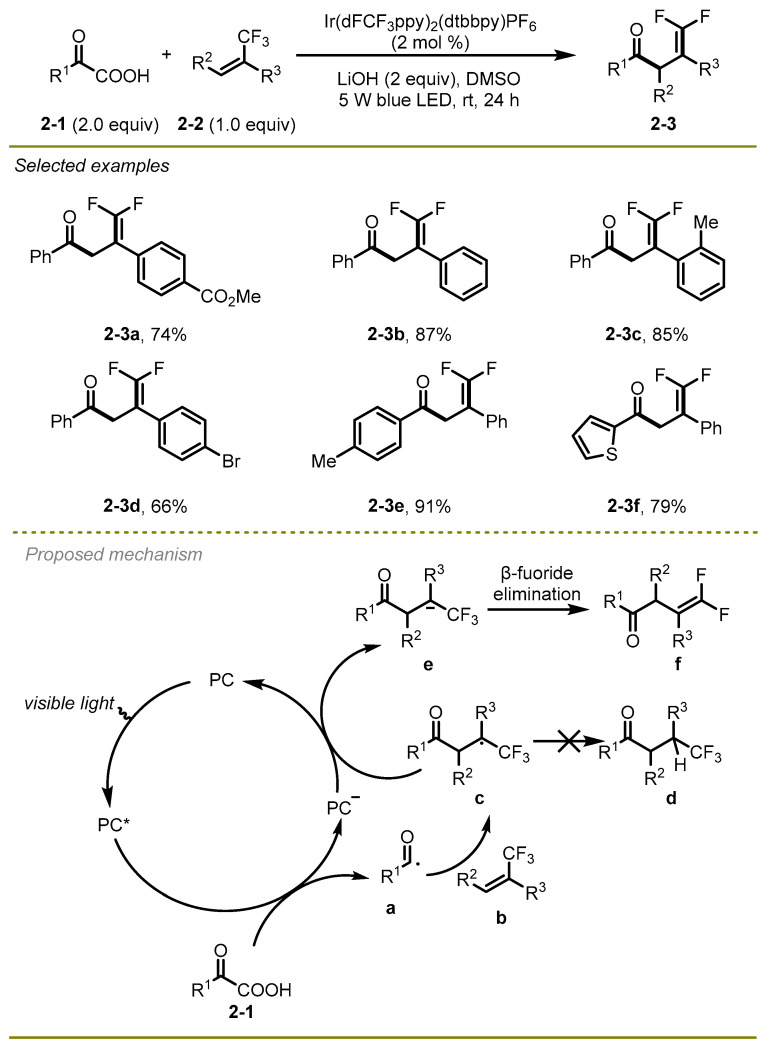
The visible-light-mediated decarboxylative/defluorinative cascade protocol.

In the next year, the Zhou group described a visible-light-mediated decarboxylative/defluorinative cascade protocol involving α-trifluoromethyl alkenes and α-keto acids, with the α-keto acids functioning as a nucleophilic acyl radical source through a radical-polar crossover process (Scheme 2) [12]. A series of electron-rich or electron-deficient substituted α-keto acids, even with a thiophenyl group (**2-3f**), and a variety of α-trifluoromethyl alkenes, such as Me and Br substituted (**2-3c**, **d**), exhibited excellent compatibilities to yield the corresponding functionalized *gem*-difluoroalkenes in moderate to good yields.

A plausible mechanism was illustrated in Scheme 2 for this transformation. In the beginning, oxidation of an appropriate α-keto acids **2-1** proceeded in the presence of the excited photocatalyst (PC*) following addition of the resulting acyl radical **a** with α-trifluoromethyl alkene **b** to deliver CF_3_-substituted alkyl radical **c**. Then it was reduced by PC^−^ via single electron transfer (SET) process to produce carbanion species **e**. Eventually, the desired *gem*-difluoroalkene **f** was afforded via β-fluoride elimination of the intermediate **e**.

**Scheme 3:**
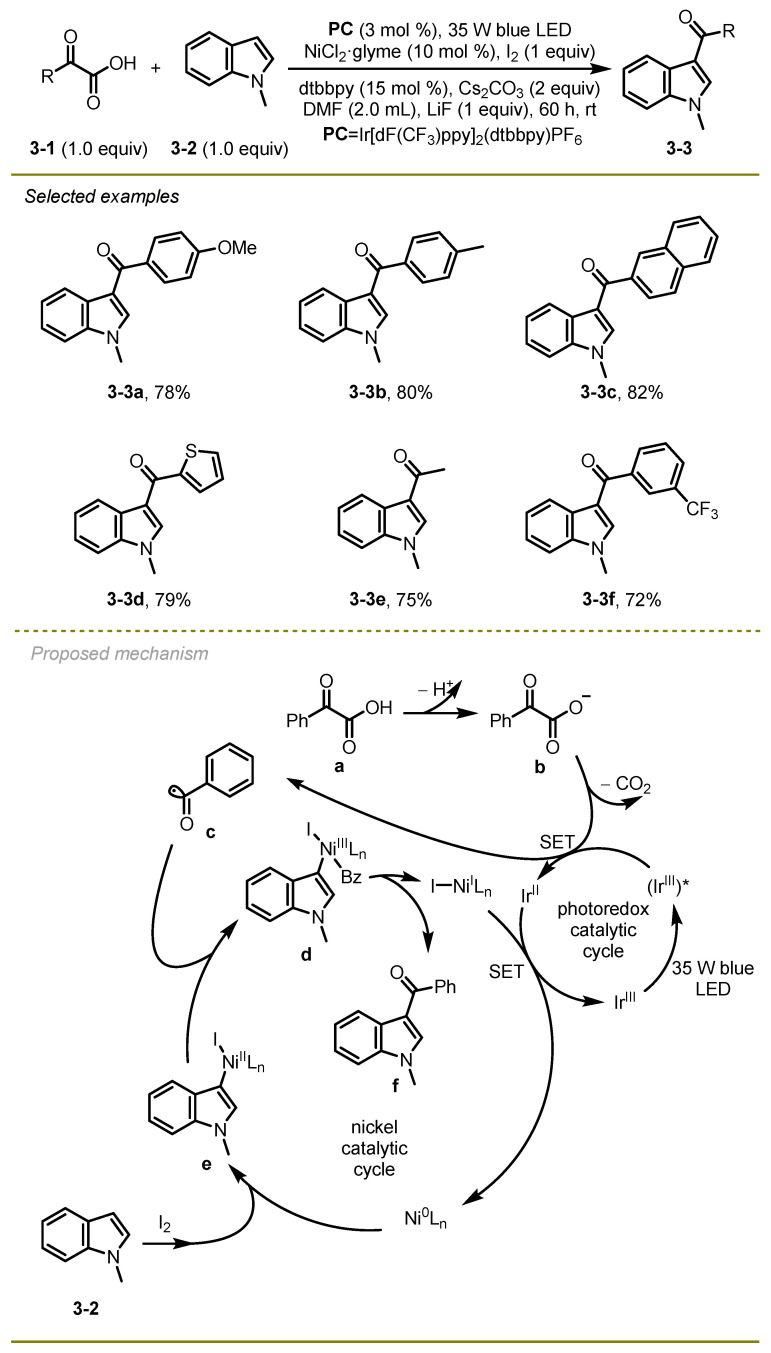
Visible-light photocatalytic acylation of diverse indoles.

A number of 3-acylindoles were prepared via visible-light photocatalytic acylation of diverse indoles with α-keto acids using Iridium/Nickel dual catalysis by Yuan and Li’s group in 2016 (Scheme 3) [13]. This acylation tolerated various functional groups, such as methoxyl (**3-3a**), and afforded the desired 3-acylindoles in moderate to good yields under mild conditions. Of note, the radical decarboxylative coupling for the synthesis of 3-acylindoles occurred without CO, strong bases, or organometallic reagents as ingredients.

From a mechanistic point of view, the reaction was initiated with the photo-excitation of photocatalyst PC followed by oxidative SET and extrusion of carbon dioxide to deliver an acyl radical **c**. Simultaneously, Indole **3-2** triggered oxidative addition of the Ni(0) catalyst in the presence of iodide to afford an intermediate **e**, which sequentially captured the radical species **c** with the formation of complex compound **d**. Finally, the corresponding 3-acylindole derivate **f** was obtained via reductive elimination, and the Ni(0) catalyst was regenerated by Ni(I) SET with Ir(II).

**Scheme 4:**
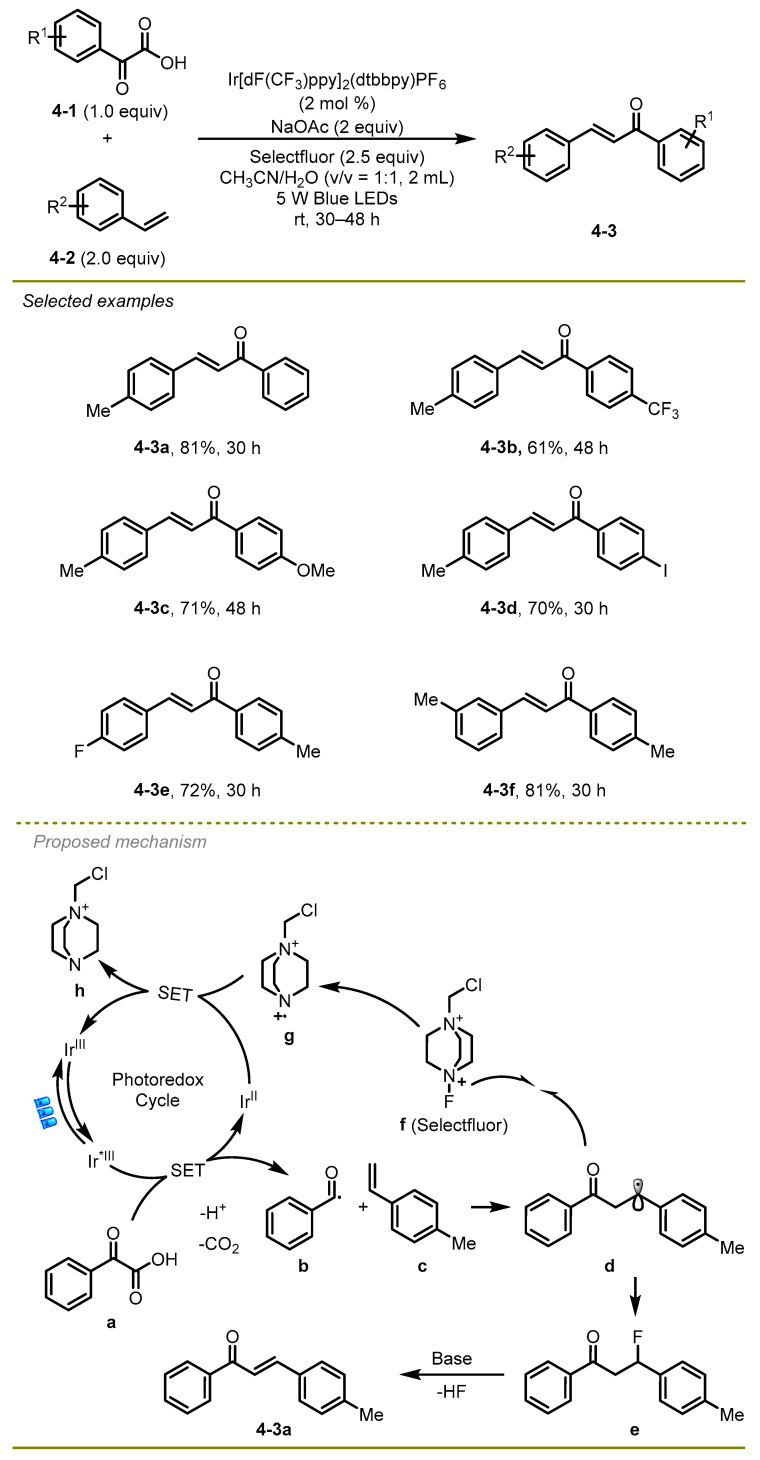
PC-catalyzed radical cross-coupling of α-keto acids.

In 2017, an elegant visible light-induced decarboxylative radical cross-coupling of α-keto acids with styrene derivatives was documented by Zhu et al., which provided an efficient protocol to generate a wide range of α, β-unsaturated ketones under mild conditions (Scheme 4) [14]. This protocol showed good tolerances to the aromatic α-keto acids and styrenes with electron-deficient or electron-rich substituents (**4-3b**, **c**). Notably, iodide-substituted α-keto acids were able to produce the desired products (**4-3d**).

Mechanistically, the excited state of photocatalyst proceeded the oxidizing SET process with α-keto acid and quickly expelled CO_2_ to initially produce benzoyl radical **b**. After radical addition, the benzoyl radical intermediate trapped the fluorine atoms from Selectfluor, generating the corresponding β-fluorinated ketones **e**. Subsequently, the elimination of HF with the assistance of NaOAc occurred to obtain the final product: α, β-unsaturated ketone **f**.

**Scheme 5:**
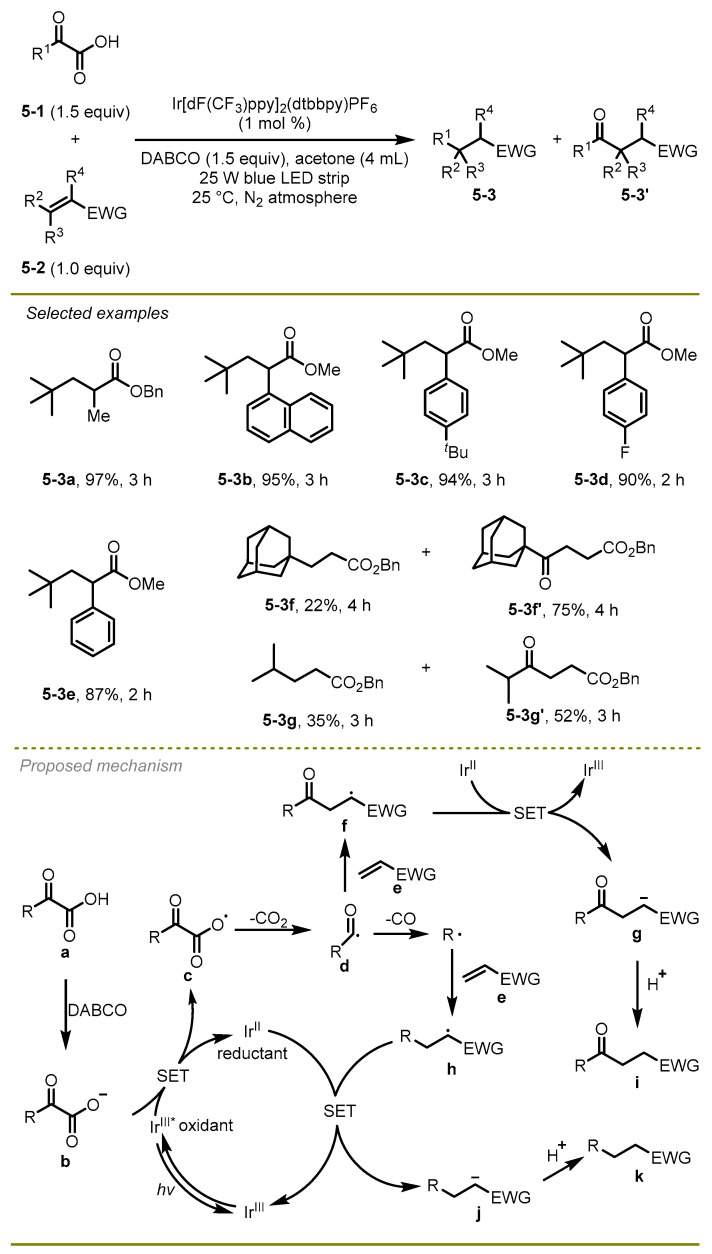
Visible light-promoted radical decarboxylation, decarbonylation, and radical addition cascade.

In 2018, Xu et al. developed a visible light-promoted radical decarboxylation, decarbonylation, and radical addition cascade of α-keto acids with electron-deficient alkenes to yield C–C bonds under mild conditions (Scheme 5) [15]. The reaction scope was wide, including sterically demanding olefins, acyclic, and cyclic unsaturated ketones, producing the target products in moderate to high yields. Notably, methyl-substituted acrylate olefins exhibited good functional group compatibilities to deliver the corresponding product at a 97% yield (**5-3a**). The synthetic utility of this pathway was demonstrated by the construction of sterically hindered tetrasubstituted carbon centers. 

A hypothesized mechanism for this radical cascade reaction was illustrated. Initially, the excited-state photocatalyst Ir[dF(CF_3_)ppy]_2_(dtbbpy)PF_6_ oxidated the anion of α-keto acid **b**, following by CO_2_-extrusion of the delivering radical **c** to produce alkyl radical **d**. Subsequently, the intermediate **d** would undergo decarbonylation and couple with an electrophilic olefin to generate a new alkyl radical **h**, which could capture an electron and then abstract a proton to form the final product **k**. Of note, in the process of the decarbonylation, acyl radical **d** theoretically easily underwent direct conjugate addition with electron-deficient olefin **e** to produce the carbonyl compounds **i**; however, this was proved to not be the primary pathway based on a variety of Stern–Volmer fluorescence quenching studies. 

**Scheme 6:**
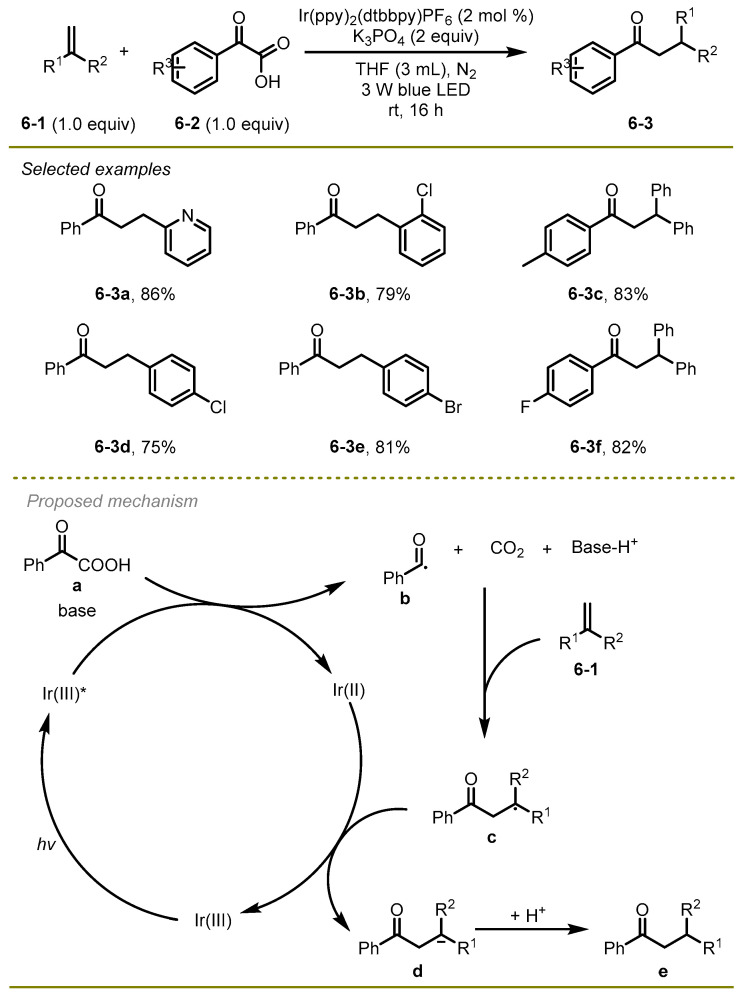
Photocatalytic decarboxylative coupling of α- keto carboxylic acids.

In 2019, the Gao, Shi, and Lei group accomplished a nice photocatalytic decarboxylative coupling of α-keto carboxylic acids and alkenes to generate a series of aryl ketones (Scheme 6) [16]. The process featured mild conditions, broad substrate scopes, and good functional group compatibilities. For example, a substrate with halogens like Cl was also well tolerated in this transformation, with a yield of 79% (**6-3b**).

A proposed mechanism was shown in Scheme 6. First, the irradiation of the Ir(III) with visible light afforded the excited photocatalyst Ir(III)*, which would oxidize α-keto acid to benzoyl radical **b** and Ir(II) with the release of CO_2_ under base conditions. Subsequently, the addition of the benzoyl radical **b** onto alkenes occurred, followed by reductive SET with Ir(II) and protonation to form the final aryl ketones **e**.

**Scheme 7:**
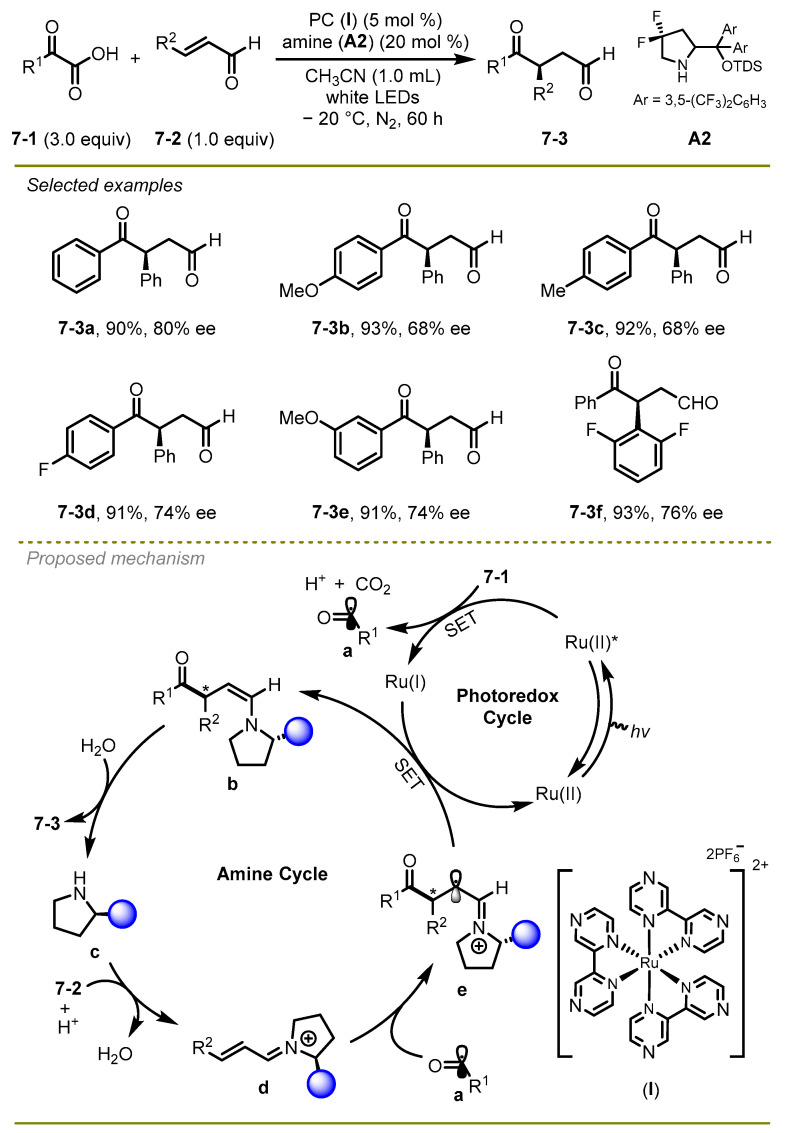
Radical hydro-acylation of enals and α-keto acids under photoredox/amine dual catalysis.

A novel and efficient strategy for the assembly of synthetically challenging 1,4-dicarbonyl compounds in an enantioselective manner was reported by Yu et al. in 2019 (Scheme 7) [17]. Notably, the authors successfully realized radical hydro-acylation of enals and α-keto acids by using photoredox/amine dual catalysis in moderate to good yields. Both of the electron-donating and electron-withdrawing ketones could undergo this transformation smoothly to yield the respective products.

Mechanistically, the reaction was triggered by Ruthenium-catalyzed oxidative decarboxylation of α-keto acids assisted by the excited-state photocatalyst and CO_2_-extrusion to produce acyl radical **a**. In parallel, an enal **7-2** was trapped by amine catalyst **c** in the presence of acid, converting into iminium cation **d**. Subsequently, a coupling between cation **d** and acyl radical **a** occurred, generating a radical species **e**, which promoted the reductive SET process along with the oxidation of Ru-cat(I) to produce enamine **b**. Eventually, the enamine **b** underwent tautomerization and hydrolysis to produce the product **7-3**.

**Scheme 8:**
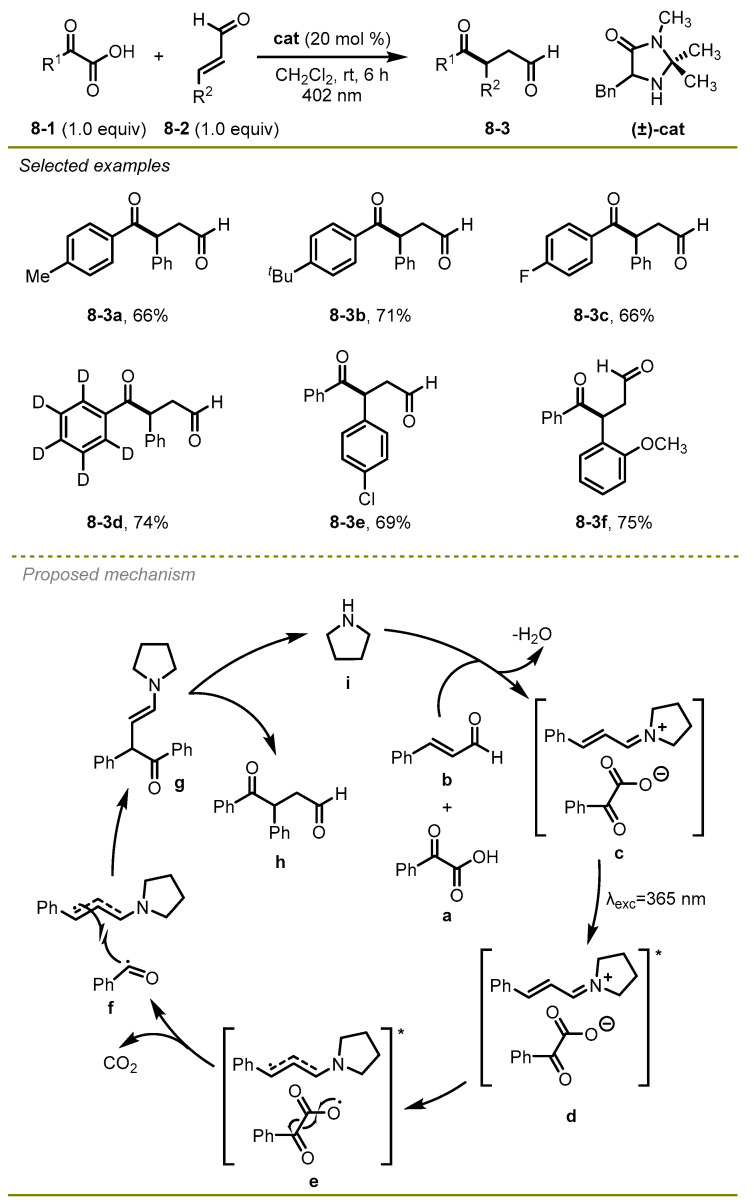
An intermolecular radical Stetter reaction of α-keto acids and aldehydes.

In 2019, Gilmour et al. developed an intermolecular radical Stetter reaction of α-keto acids and aldehydes to generate 4-keto aldehydes with high efficiency and good chemoselectivity (Scheme 8) [18]. Of note, the authors found that radical-radical coupling lagged behind single electron transfer and rapid decarboxylation in the approach. Both α-keto acids and unsaturated aldehyde derivatives with *para* or *ortho* substituents on the phenyl ring were suitable substrates to conduct this reaction.

A plausible mechanism for radical pathway is illustrated in Scheme 8. First, the excited state of close ion pair **d** could undergo a single electron transfer and intersystem crossing to deliver radical species **f**. Subsequently, the radical pair **f** promoted radical–radical cross-coupling to furnish intermediate **g**. Eventually, the desired products were obtained via hydrolysis of the enamine **g**.

**Scheme 9:**
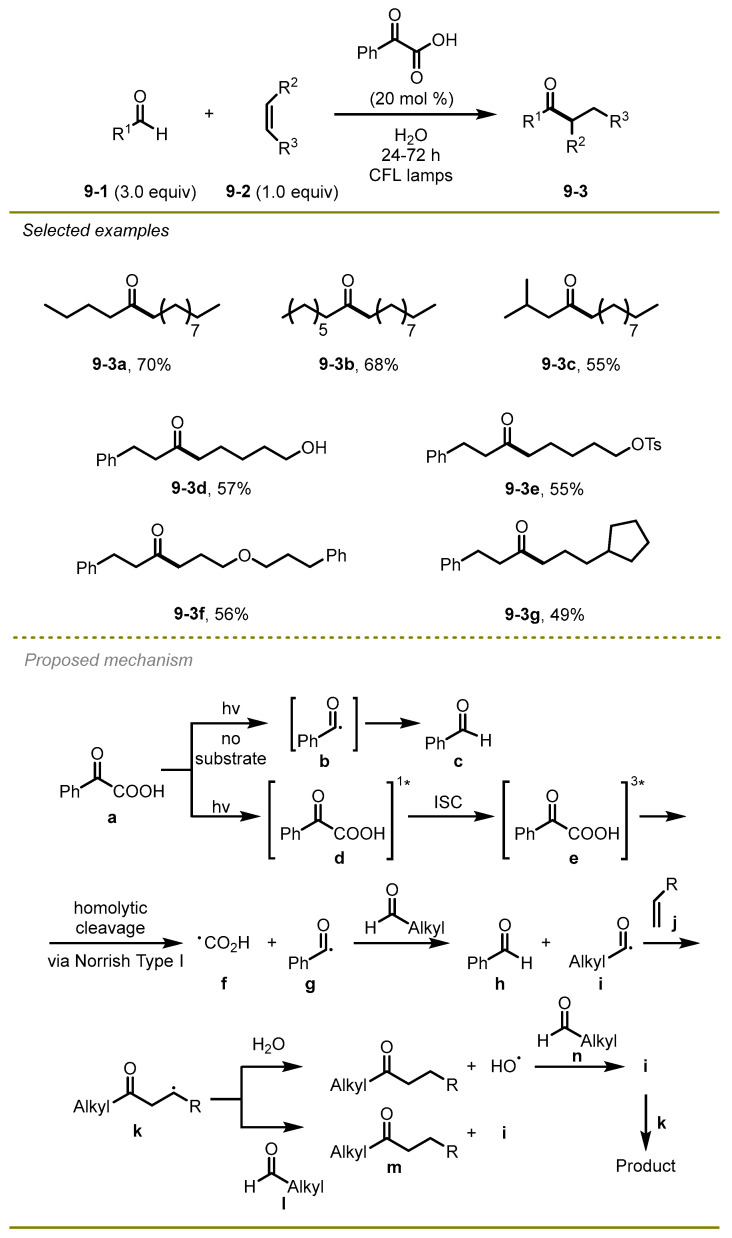
Visible-light photocatalytic hydroacylation of unactivated electron-rich alkenes in water.

In 2020, Kokotos et al. disclosed a green, metal-free, and efficient method that directly transferred aldehyde into ketone with the formation of a C–C bond via visible-light photocatalytic hydroacylation of unactivated electron-rich alkenes in water without a metal catalyst (Scheme 9) [19]. Of note, the strategy employed phenyl-substituted α-keto acids as a novel photo-initiator to efficiently achieve different kinds of ketones under the irradiation of household lamps in water. The strategy exhibited good compatibility to aliphatic aldehydes with various functional groups, yielding the expected products in moderate to excellent yields.

The plausible reaction mechanism involved the formation of phenyl-containing α-keto acids via irradiation excitation and intersystem crossing, which triggered homolytic cleavage of the carbon-carbon single bond to produce a benzoyl radical **g**. Then, the benzoyl radical reacted with the aldehyde, delivering the first acyl radical **i**, followed by the addition of an electron-rich alkene to form radical intermediate **k**. Ultimately, a hydrogen atom was abstracted from water or the other aldehyde to the radical **k,** providing the desired product.

### 2.2. Acylative Difunctionalization of Alkenes

**Scheme 10:**
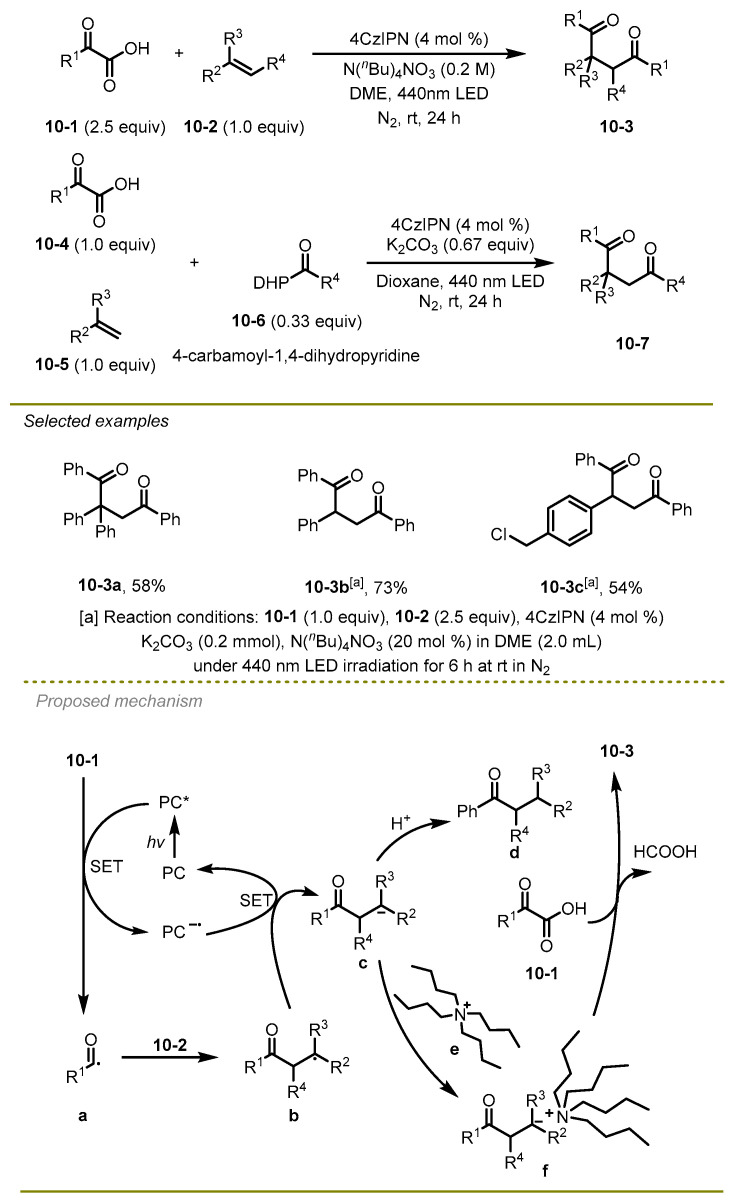
A straightforward, metal-free, and regioselective catalytic synthesis of the unsymmetric or symmetric 1,4-dicarbonyl compounds.

Distinguished from the above-mentioned methods, which only realized mono-carbonylation of alkenes, in 2021, the Wu group documented a straightforward, metal-free, and regioselective catalytic synthesis of the unsymmetric or symmetric 1,4-dicarbonyl compounds through 1,2-dicarbonylation of alkenes (Scheme 10) [20]. Notably, the protocols employed tetrabutylammonium cation N(n-Bu)_4_^+^ to activate α-keto acids undergoing electrophilic addition instead of protonation to achieve the synthesis of 1,4-dicarbonyl compounds. It is the first study to produce unsymmetric 1,4-dicarbonylation via adjusting the reducing and electrophilic capabilities of the acyl precursor. The aryl group of styrene, substituted by different electronic effects groups or larger steric hindrance substituents, could successfully produce the corresponding 1,4-diketones.

A plausible mechanism was illustrated. First, α-keto acids **10-1** were oxidized by the excited photocatalyst 4CzIPN* via a SET to generate the reductive PC^−^ and acyl radical **a**, which were added onto the styrene species **10-2** and resulted in the formation of alkyl radical **b**. Subsequently, the PC^−^ would mediate reductive SET of alkyl radical **b** to afford the ground state of PC and alkyl anion **c**. It is worth mentioning that the authors selected tetrabutylammonium cation (N(n-Bu)_4_^+^) to stabilize anion **c** and yield the salt **f** instead of being protonated, thereby generating mono-ketone **d**. Finally, compound **f** was trapped by α-keto acids as ketone carbonyl precursors to achieve the desired products via β-carbonylation.

**Scheme 11:**
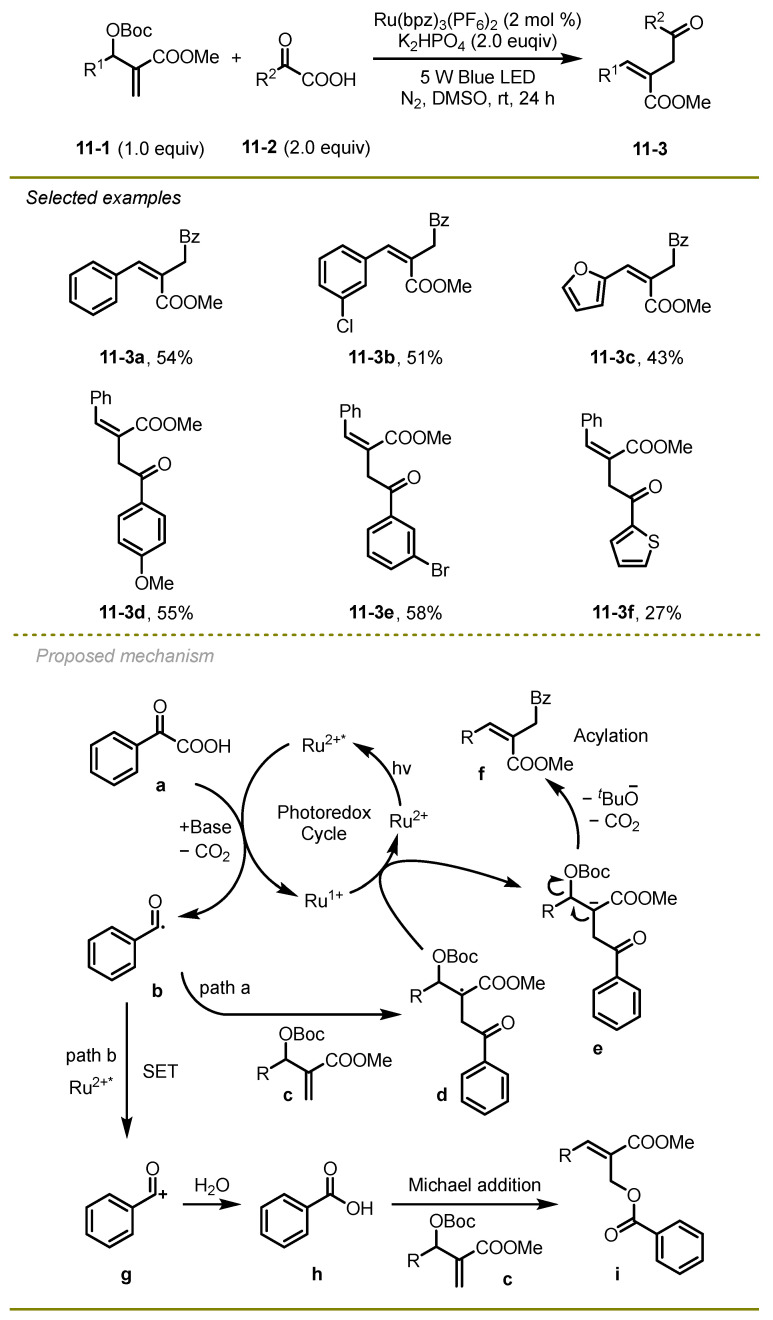
A photocatalytic decarboxylative radical coupling of α-keto acids with Baylis–Hillman derivatives.

Further extension of visible-light photocatalytic decarboxylative radical coupling of α-keto acids with Baylis–Hillman derivatives was disclosed by Li and Xu et al. in 2021, which could effectively yield a variety of acyl-substituted trisubstituted olefins (Scheme 11) [21]. Notably, the different electronic effects on aryl or heterocyclic substituted α-keto acids and Baylis–Hillman derivatives were tolerated very well when seeking to obtain the corresponding products at an acceptable yield.

From a mechanistic point of view, the excited state of photocatalysis Ru*^2+^ proceeded SET with α-keto acid to deliver reductive Ru^1+^ and acyl radical **b** via decarboxylation, which immediately added to the double bond of Baylis–Hillman carbonate **c** with the generation of the alkyl radical **d**. Subsequently, the alkyl radical **d** was reduced by Ru^1+^ to furnish the ground state of Ru^2+^ and the alkyl anion **e**, followed by an elimination to yield the target product **f** (path a). It is worth mentioning that the acyl radical **b** could be further oxidized by Ru*^2+^ to afford cation species **g**, which would undergo a protonation with water to deliver carboxylic acid **h**. A Michael addition of **h** and Baylis–Hillman carbonates **c** produced by-product **i** (path b).

**Scheme 12:**
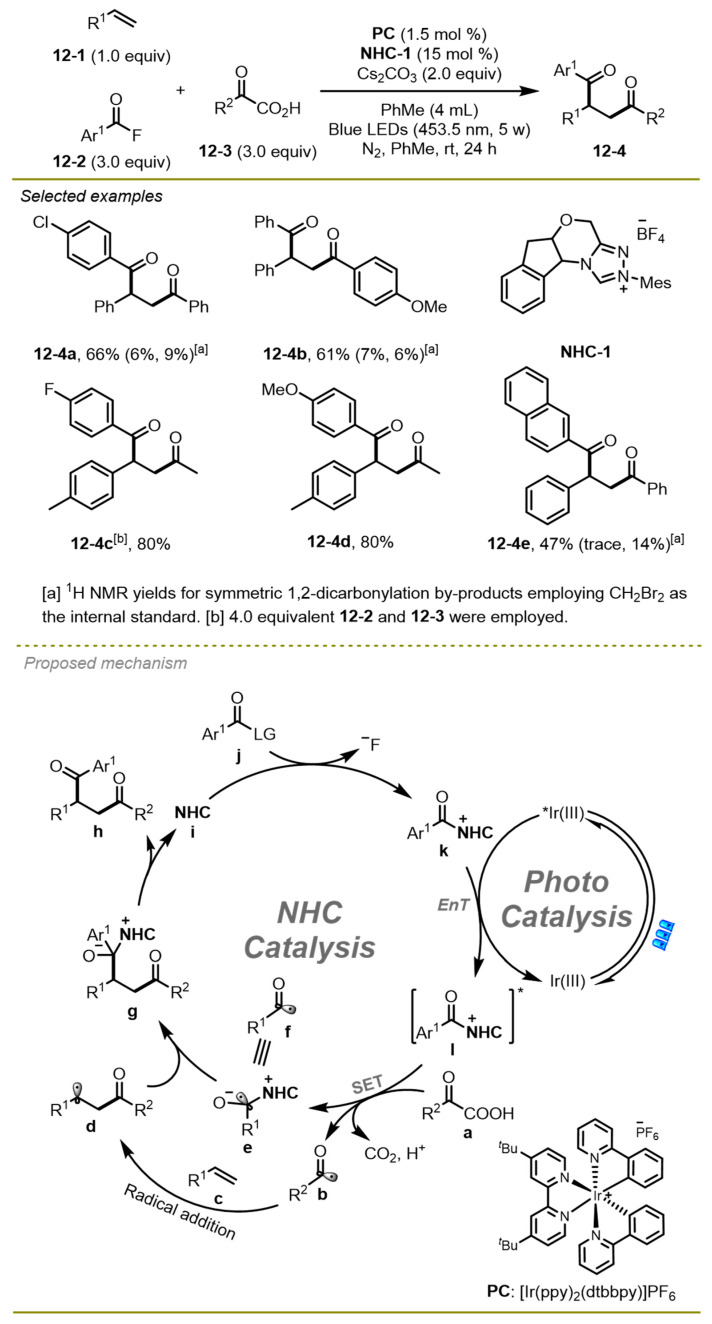
NHCs and photoredox co-catalyzed three-component unsymmetric radical 1,2-dicarbonylation of α-keto acids.

In 2022, Zheng et al. also realized unsymmetric radical 1,2-dicarbonylation of alkenes with α-keto acids with the aid of aroyl fluorides (Scheme 12) [22]. Notably, the process was activated by NHCs catalyst, and photo-redox co-catalyzed a three-component catalyst to synthesize selective desired products with excellent functional group compatibilities. The different electronic and steric effects of α-keto acids and aroyl fluorides on aryl exhibited good tolerance in this transformation.

As for the mechanism, a radical process was proposed. The acyl fluoride **j** was trapped by NHCs to generate acylazolium intermediate **k** and then gave rise to the excited species **l** in the presence of the excited-state of [Ir(ppy)_2_(dtbbpy)]PF_6_, which could undergo SET with α-keto acid **a** to obtain NHC-stabilized ketyl radical intermediate **e** and acyl radical **b**. The latter added to the double bond of olefin **c**, along with the resulting alkyl radical **d** coupling with the complex radical **e** as well as the dissociation of NHCs-product complex to produce 1,4-dicarbonyl compound **h**.

**Scheme 13:**
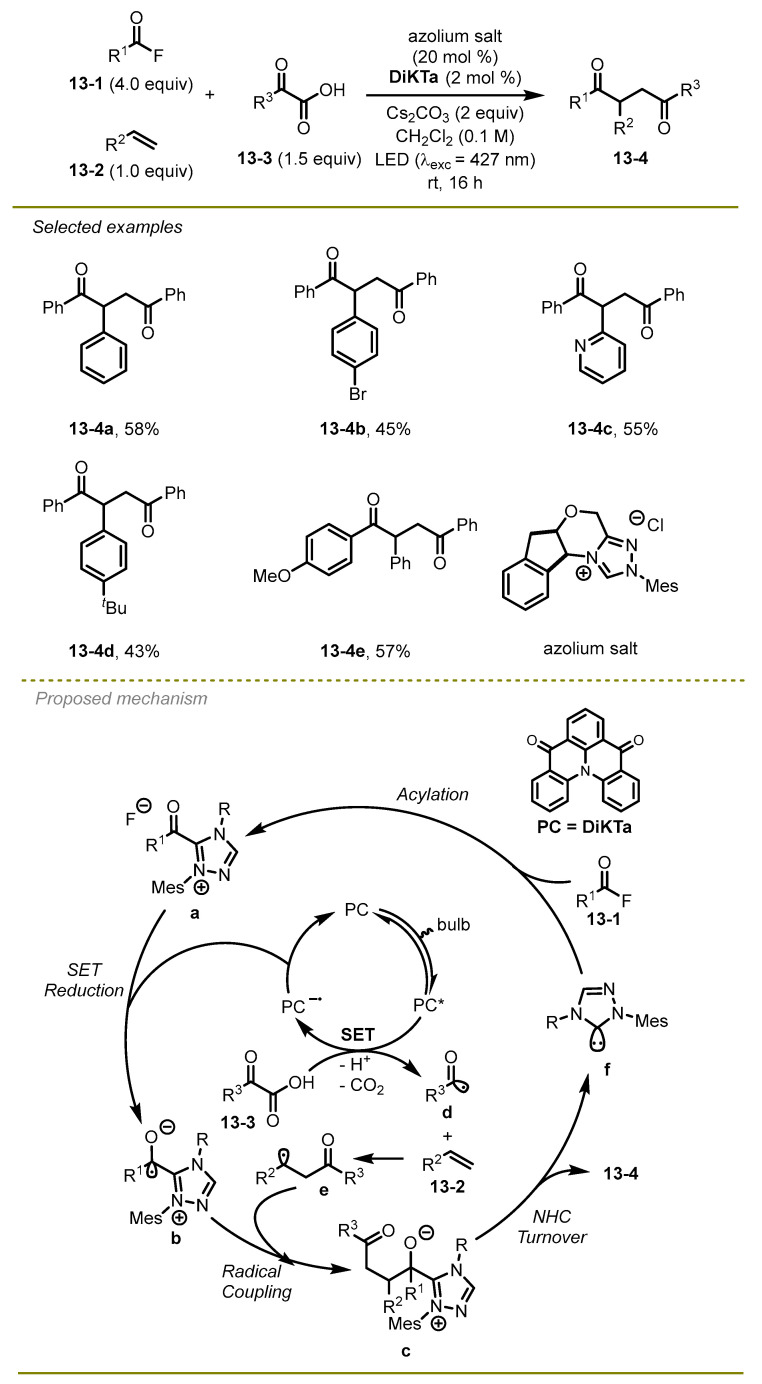
Dual NHC/photoredox catalytic synthesis of 1,4-diketones.

In the same year, Smith and Zysman-Colman et al. disclosed the organo-photocatalyst DiKTa. NHC dual catalysis could be employed in the assembly of unsymmetric 1,4-dicarbonyl compound (Scheme 13) [23]. Alkene derivatives such as styrene showed good tolerances to these kinds of substituents. The benzoyl fluorides with either electron-donating or electron-withdrawing substituents were readily converted into the target compounds in high yields. In contrast to Zhang’s work, the process took advantage of not using a transition metal in the catalytic system, although certain products would gain better yields under previous conditions.

The plausible mechanism for the radical reaction was shown. The authors proposed that benzoyl fluoride promoted the acylation of the in situ generated NHC to produce intermediate **a**. The excited state of photocatalyst DiKTa oxidized α-keto acid to obtain the anion of PC and an acyl radical **d**, which could be trapped by styrene to deliver stable alkyl radical **e**. Subsequently, the ketyl intermediate **b**, which was reduced by the anion of PC, underwent radical-radical coupling with alkyl radical **e** to produce intermediate **c**, followed by dissociation to yield the target 1,4-diketone product **13-4**.

**Scheme 14:**
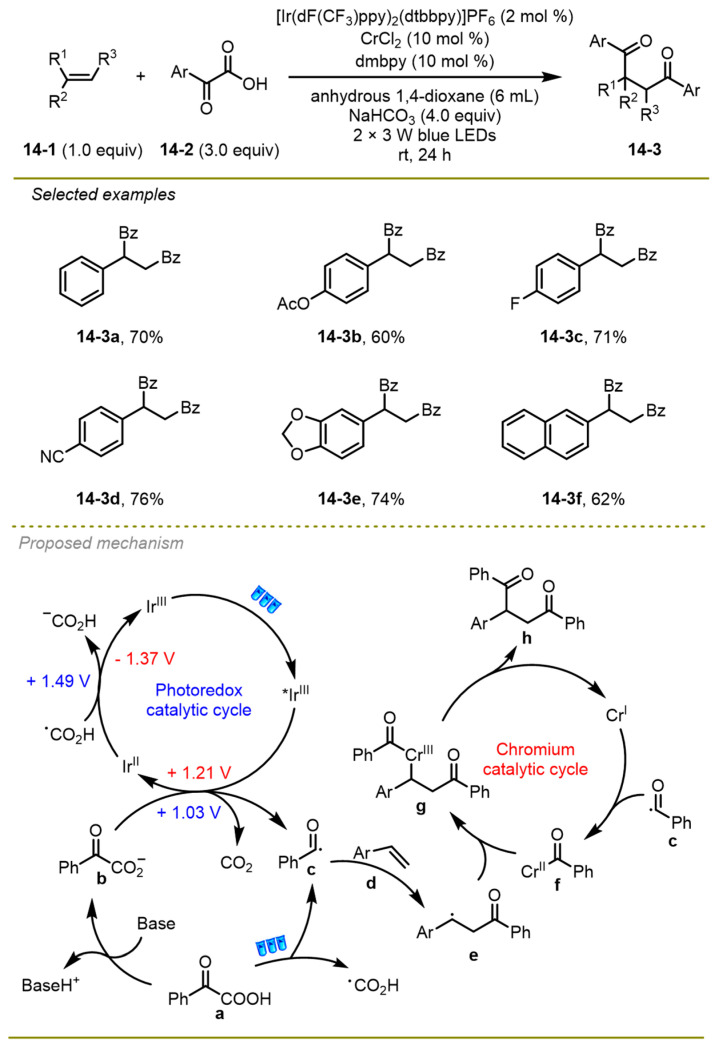
The diacylation of alkenes in the presence of dual photoredox/chromium catalysis.

Instead of the widely used palladium and nickel catalysis, Qi and Cheng et al. reported that a wide range of alkenes could also be activated by chromium catalysis via visible-light photocatalytic diacylation of alkenes to obtain the corresponding diones in 2022 (Scheme 14) [24]. Notably, translating diones into a series of valuable heterocycles showed the practicality of this synthetic protocol. This reaction tolerated substituents at different positions on the phenyl-substituted olefins in moderate to good yields.

From a mechanistic point of view, the excited-state Ir(III)* catalyst was trapped by α-oxocarboxylate **b**, which was given by the deprotonation of α-keto acids, undergoing SET to afford acyl radical **c** and the reduced photocatalyst along with CO_2_-extrusion. The former also could be delivered through the fragmentation of α-keto acids under visible light irradiation. Subsequently, acyl radical **c** was added onto double bond of alkene to generate a nucleophilic alkyl radical **e**, which was rapidly trapped by Cr(I) species to yield a complex compound **f** in the meantime. Then, the intermediate **e** underwent the coupling reaction with complex species **f**, resulting in a stable Cr(III) complex **g**. The final 1,4-dione product could be formed via the reductive elimination of the intermediate **g**.

**Scheme 15:**
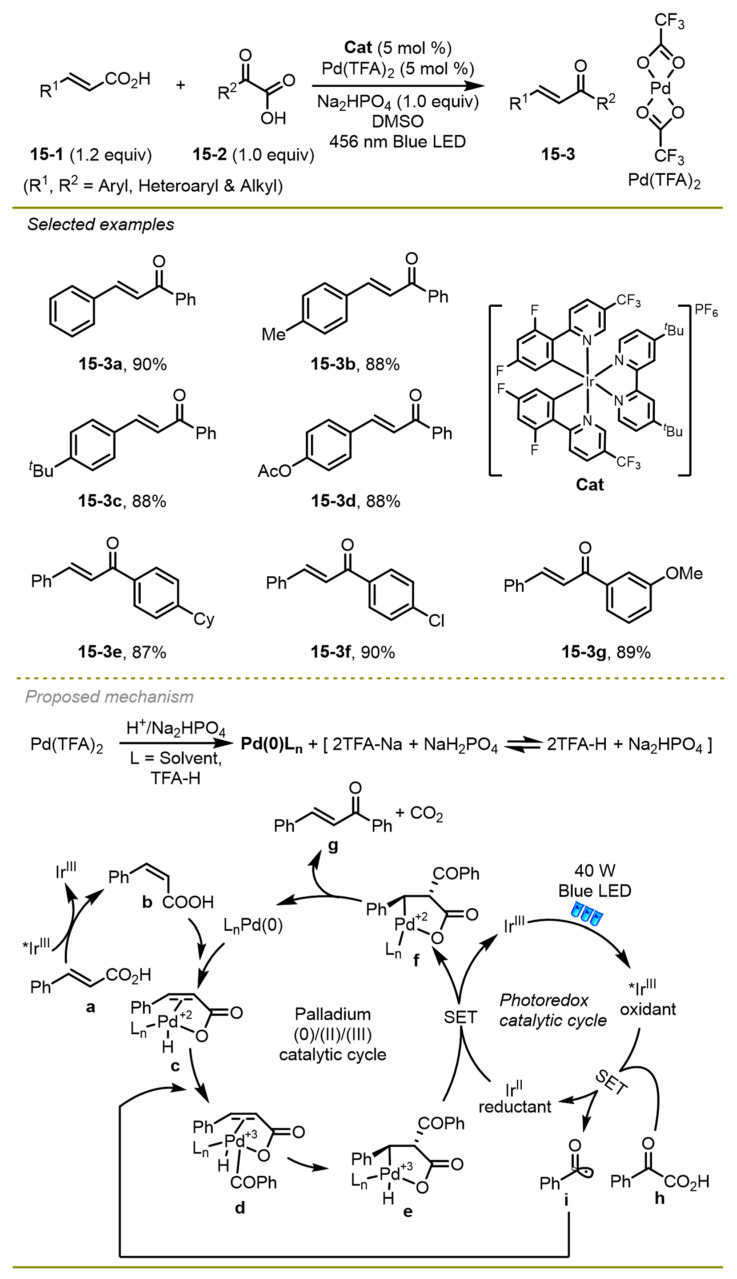
Doubly decarboxylative C_sp_^2^-C_sp_^2^ cross-coupling reaction.

Compared with addition onto alkenes to accomplish its difunctionalization, Ghosh et al. presented a doubly decarboxylative cross-coupling reaction between α-keto acids and α, β-unsaturated acids to generate a series of α, β-unsaturated ketones (Scheme 15) [25]. The strategy used iridium/palladium dual catalysis to proceed C_sp_^2^-C_sp_^2^ cross-coupling reaction with excellent selectivity and broad scope. Aromatic α, β-unsaturated acid and aromatic α-keto acid could bear different electronic effects on the phenyl ring, obtaining the target products in excellent yield.

Mechanistically, the SET from α-keto acid to the excited iridium (III)* catalyst formed the reductant Ir(II) catalyst and acyl radical **i** with the release of CO_2_. At the same time, trans-cinnamic acid **a** could also change into its *cis* isomer, taking advantage of the photo-excited energy that followed the oxidative addition with palladium(0) catalyst and afforded Pd(II)-intermediate **c**. Then, the intermediate **c** was one-electron oxidated by acyl radical **a**, forming a new Pd(III)-intermediate **d**, followed by β-migratory insertion of the acyl group and one-electron reduction with the reductant Ir(II) species to achieve Pd(II)-intermediate **f**. Finally, the resulting intermediate **f** underwent a synergetic decarboxylative demetallation and gained the desired product: α, β-unsaturated ketone.

**Scheme 16:**
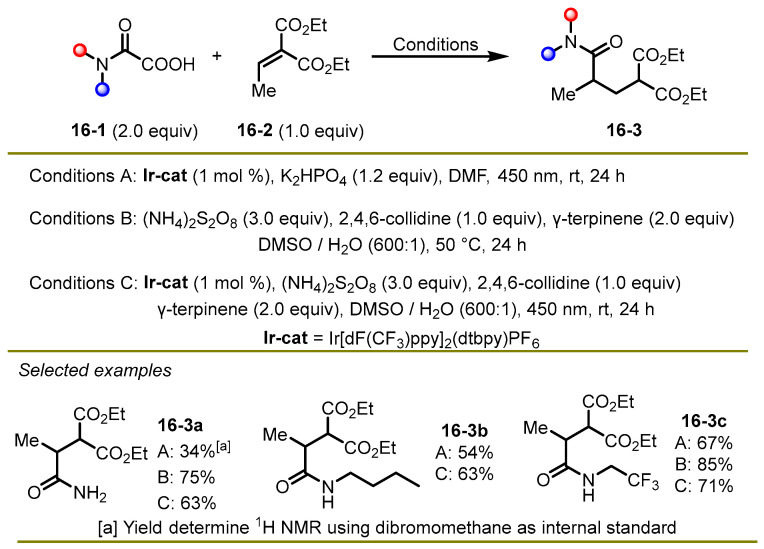
Direct decarboxylative Giese amidations under three different conditions.

In 2023, the Lee group accomplished intermolecular direct decarboxylative Giese amidation from α-keto acids via radical conjugate addition reactions, which provided a concise and direct approach to synthesize 1,4-difunctional compounds (Scheme 16) [26]. Interestingly, the authors compared the experimental outcomes under three different conditions, containing both visible light-promoted and thermal initiation, which were complementary and flexible in the range of substrate compatibility.

The authors documented three conditions: photocatalytic reductive quenching cycle (conditions A); metal- and light-free (conditions B); and photocatalytic oxidative quenching cycle (conditions C). In general, conditions B and C both exhibited superior yield in contrast to condition A in the synthesis of secondary amides. However, for the synthesis of tertiary amides, condition A could prevent the formation of dealkylated side products to produce the desired tertiary amides, while the dealkylated secondary amides were delivered in the oxidative conditions B and C.

**Scheme 17:**
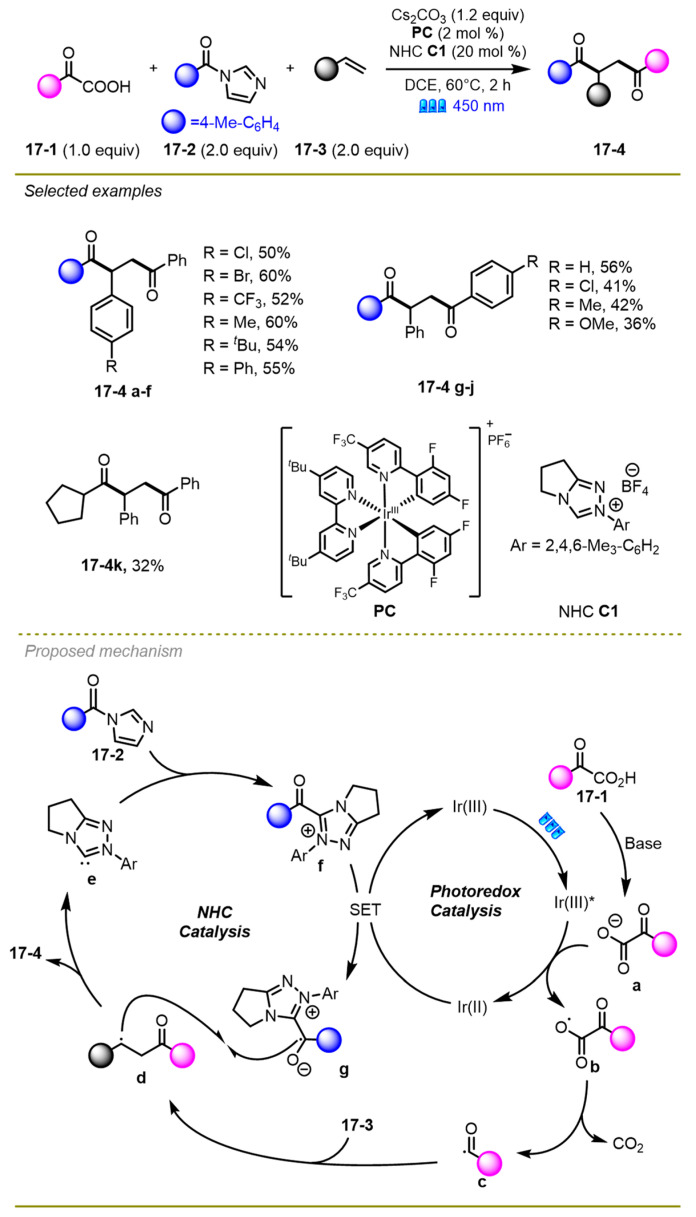
Regioselective unsymmetric bis-acylation of olefins.

In 2023, Zhang et al. disclosed a regioselective unsymmetric bis-acylation among olefins, α-keto acids, and acyl imidazoles through photoredox/Iridium dual catalysis (Scheme 17) [27]. A variety of substituents with different electronic effects, such as the challenging alkyl acyl imidazoles (**17-4k**), were tolerated well in acceptable yields under mild conditions. Moreover, the synthesis of significant ploy substituted heterocycles from 1,4-diketones proved the synthetic utility of this strategy. 

As for the mechanism, first, α-keto acid dissociated a proton in the presence of the base to obtain α-oxocarboxylate, following by oxidative SET with the excited state of the Ir(III)* catalyst and the decarboxylation process to give an acyl radical **c**. Subsequently, the acyl radical could be trapped by styrene **17-3** converting into benzyl radical **d**. The cation **f** was reduced by Ir(II) to give acyl azolium species **g**. These two radicals finally encountered, producing the desired product **17-4** through a cross-coupling reaction along with the release of carbene catalyst. 

## 3. Decarboxylative Cyclic Acylation of α-Keto Acids with Alkenes

Keto-containing cyclic architectures such as indolones, quinolinones, phenanthridines, coumarins, chromones, benzothiophenes, and so on are a highly valuable class of molecules involved in various kinds of well-known organic compounds of industrial and pharmacological significance, such as optics, electronics, and material sciences as well as anticancer, antifungal, and antibacterial agents, which renders the research on facile and efficient construction of such complex scaffolds to be a hot topic and extremely attractive for further exploration. A variety of acylated cyclic molecules could be obtained using α-keto acids via the decarboxylative cyclization/acylation or acylation/cyclization with acyl radicals as intermediates.

**Scheme 18:**
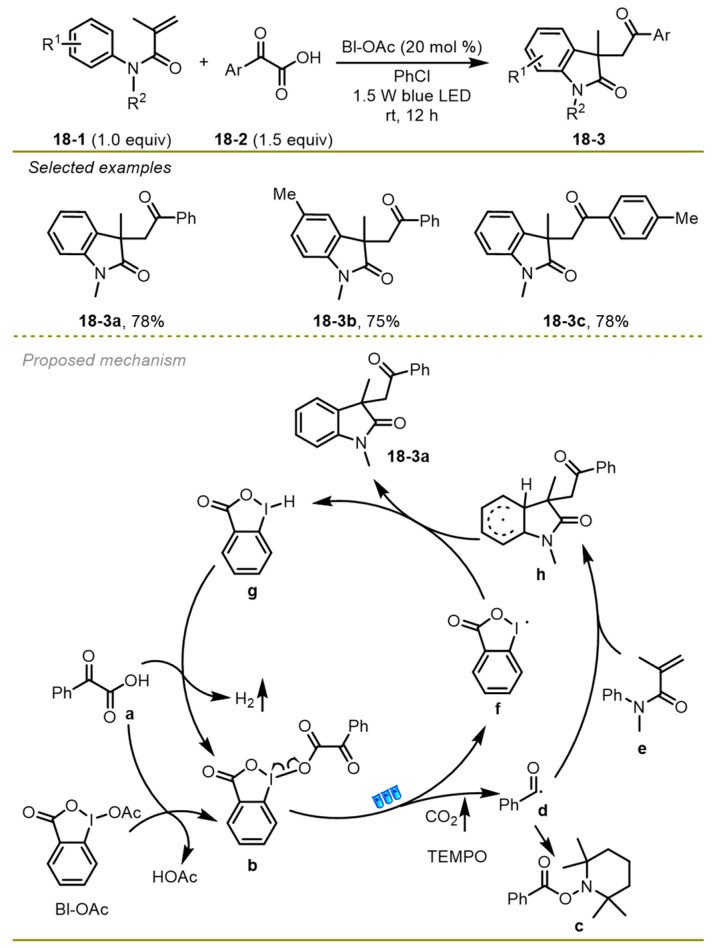
Visible light-induced direct decarboxylative arylation of acryl-incorporated amides.

In 2016, the Wang group accomplished a visible light-induced photocatalyst-free direct decarboxylative acyl-arylation of acryl-incorporated amides with α-keto acids driven by a hypervalent iodine reagent, which provided an efficient entry to construct a variety of oxindoles (Scheme 18) [28]. The strategy was compatible with a wide range of substituted groups on the benzene rings of amides, such as Me or MeO groups (**18-3c, d**), and furnished the target oxindoles in good yields at room temperature. 

Mechanistically, α-keto acids would undergo transesterification with hypervalent iodine reagent BI–OAc to deliver complex intermediate **b**, which was followed by homolytic cleavage to produce both iodanyl radical **f** and benzoyl radical **d** with the release of CO_2_ under the irradiation of blue LED. Subsequently, benzoyl species **d** added onto amide **e** and gave access to a more stable radical **h** through intramolecular radical cyclization, followed by a hydrogen atom transfer from radical **h** to iodanyl radical **f** to yield the desired product **i**.

**Scheme 19:**
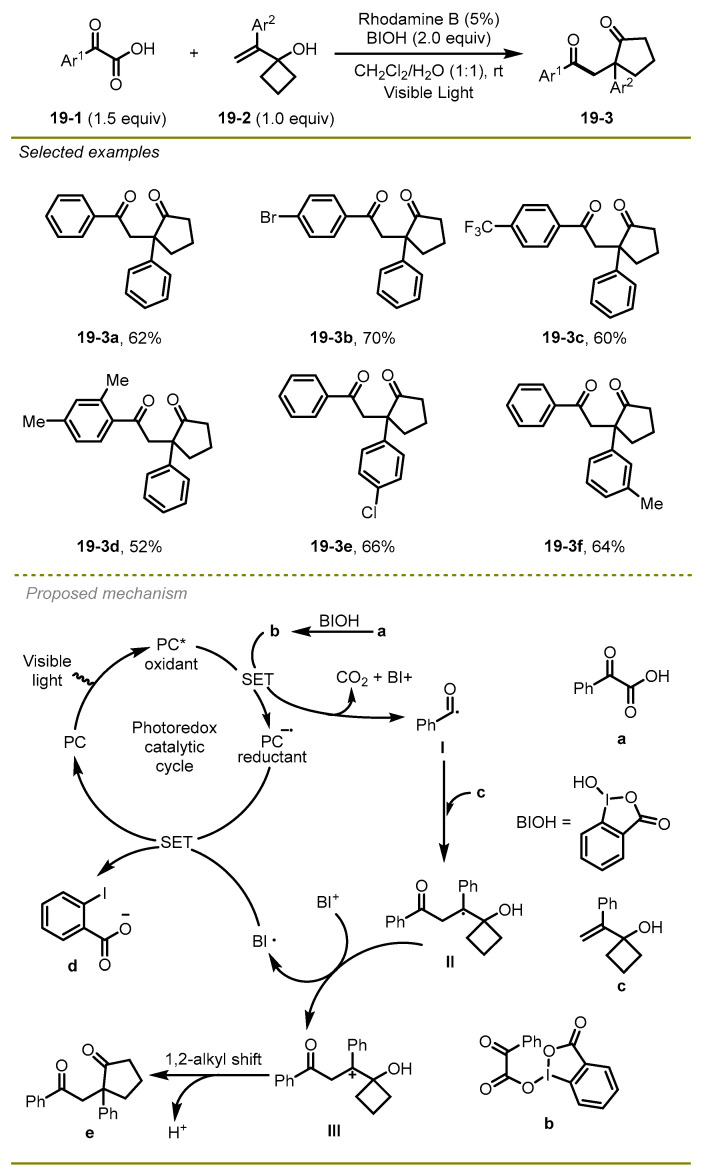
Decarboxylative acylation and ring expansion of vinylcyclobutanol.

In the next year, Duan et al. disclosed that α-keto acids and vinylcyclobutanol could be activated by metal-free photocatalysis to achieve the corresponding 1,4-dicarbonyl compounds via decarboxylative acylation and ring expansion (Scheme 19) [29]. Notably, the process employed a hypervalent iodine reagent as an oxidant under mild conditions with a good broad scope. This strategy had good tolerance to a range of α-keto acid substrates with electron-deficient or -rich substituents, such as bromo and trifluoromethyl on the aryl ring (**19-3b**, **c**), affording the corresponding products in moderate to good yields.

In view of the proposed radical mechanism, first, a reductive quenching process of the excited state PC* with the hypervalent iodine intermediate **b**, which was generated by the combination between α-keto acids and hypervalent iodine reagent BIOH, led to the formation of acyl radical **I** and the reductant of PC^−^ and the extrusion of CO_2_. The addition of the vinylcyclobutanol with acyl radical **I** followed the oxidation of the affording alkyl radical **II** to form the corresponding carbocation intermediate **III**. Subsequently, intermediate **III** was converted into the desired product **e** through a 1,2-migration of alkyl along with the dissociation of a proton.

**Scheme 20:**
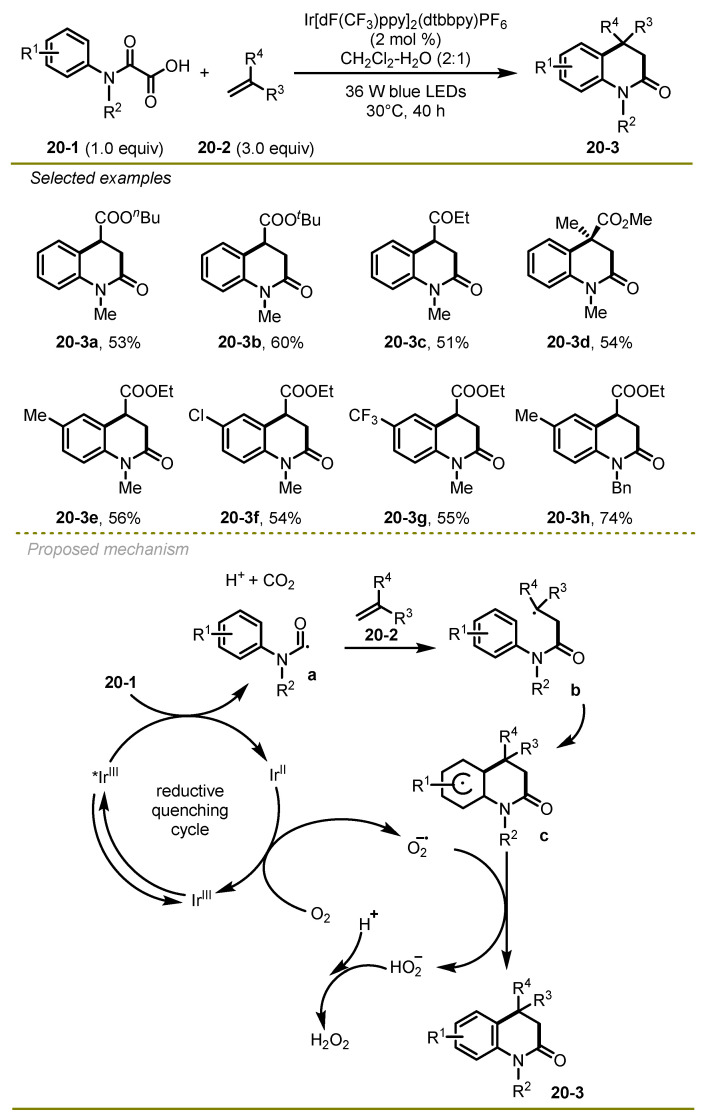
Visible-light-promoted radical addition/cyclization/aromatization cascade process.

Considering the significance of nitrogen-containing heterocycles, Feng et al. developed a new visible-light-promoted decarboxylative approach to access 3,4-dihydroquinolin-2(1H)-ones via a radical addition/cyclization/aromatization cascade process in 2018 (Scheme 20) [30]. The transformation scope was wide and included electrophilic alkenes, such as *n*-butyl acrylate and ethyl vinyl ketone, and a series of α-keto acids with various electronic effects on the aryl or N atom, such as the Me group.

As for the mechanism, the excited Ir(III)* photocatalyst underwent single-electron SET with α-keto acids **20-1** to obtain Ir(II) species, CO_2_, and carbamoyl radical **a**, which could sequentially promote intermolecular nucleophilic addition and intramolecular homolytic aromatic substitution to form cyclohexadienyl species **c**. Ir(II) species was oxidated by the O_2_ to obtain an oxygen radical anion and regenerated the ground state Ir(III) photocatalyst, finishing the catalytic cycle. At the same time, a hydrogen atom was abstracted from cyclohexadienyl radical **c** by the oxygen radical anion to yield the desired 3,4-dihydroquinolin **20-3**.

**Scheme 21:**
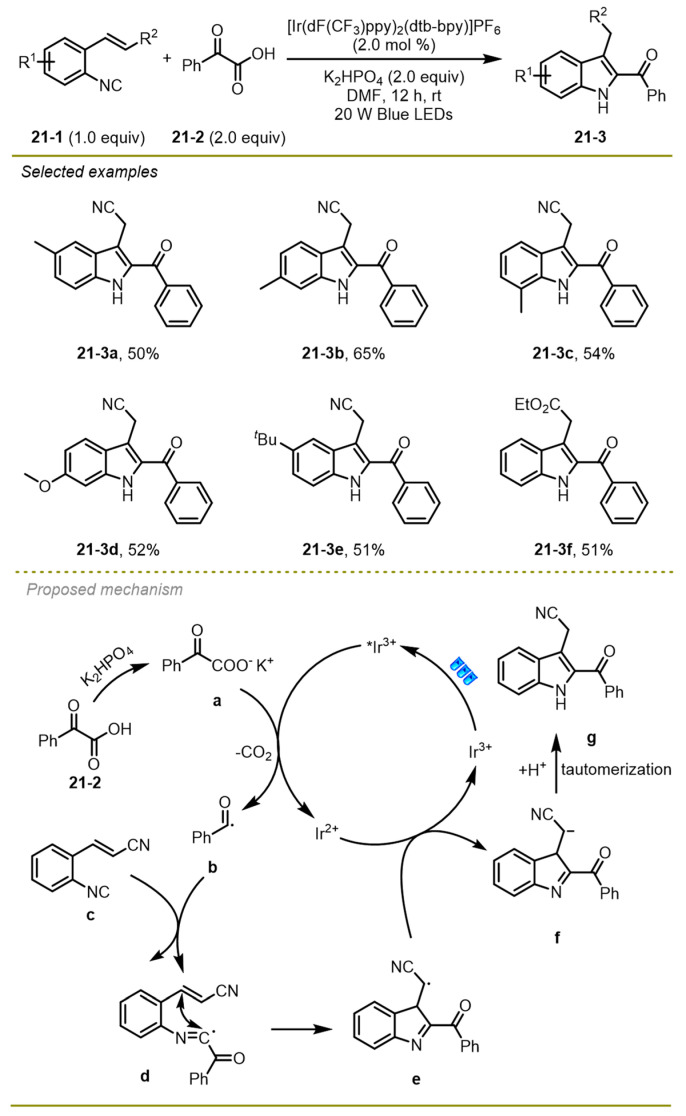
Visible-light photocatalytic decarboxylative cyclization.

In 2020, Zhang and Yao et al. described a novel synthesis of a series of 2-acylindoles derivatives via visible-light photocatalytic decarboxylative cyclization between 2-alkenylarylisocyanides and α-keto acids (Scheme 21) [31]. 2-Alkenylarylisocyanides with electron-deficient or electron-rich aryl substituents, such as alkyl and methoxyl (**21-3 a-d**), could undergo this reaction smoothly to provide the products in moderate to good yields. 

Mechanistically, first, the anion generated from base-mediated deprotonation of α-keto acid could undergo single electron transfer oxidation and decarboxylation with the excited Ir(III)* species to construct acyl radical **b** and the Ir(II) photocatalyst, which added onto the double bond of 2-alkenylarylisocyanide **c** to yield an intermediate **d**, which would undergo a 5-*exo*-*trig* cyclization to afford a benzyl radical **e**. The radical was reduced by Ir(II) through a reductive SET process, generating benzyl anion **f** along with the formation of ground state of Ir(III) species. Finally, the former was converted into 2-acylindole **g** through a protonation and isomerization process.

**Scheme 22:**
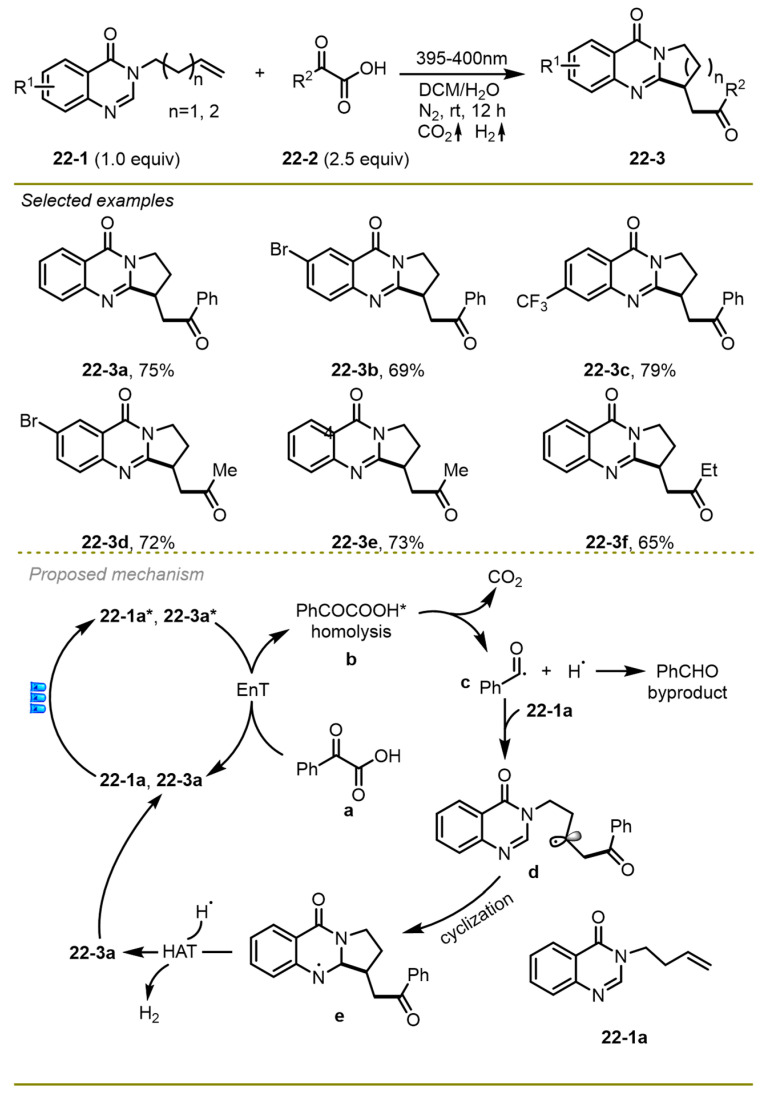
Visible light-promoted cascade radical decarboxylative acylation/cyclization process.

A visible light-promoted cascade radical decarboxylative acylation/cyclization process of unactivated alkenes with readily available α-keto acids was disclosed by the Ji group in 2021, which provided an efficient and green strategy to produce a considerable number of quinazolinone derivatives in the absence of photocatalysts and external oxidants (Scheme 22) [32]. Notably, the protocol employed a novel pathway for gaining an acyl radical from α -keto acids through a self-catalyzed energy transfer process compared with the traditional strategy. A wide range of quinazolinones and α-keto acids with a variety of functional groups, such as Br, CF_3_, and Et, on the benzene ring worked smoothly to provide the target products.

From the proposed reaction pathway, first, the excited state of substrate **22-1a** could undergo an energy transfer process with ground-state benzoylformic acid **22-3a** employed as a photosensitizer with the formation of excited species 22-3a*. The decarboxylation of the excited PhCOCOOH* followed the addition of the resulting benzoyl radical with electron-rich alkenes, producing intermediate **d**. An intramolecular cyclization could then deliver a more stable radical intermediate **e**. Finally, the hydrogen atom transferred from intermediate **e** to hydrogen radical, yielding the corresponding product with the release of H_2_.

**Scheme 23:**
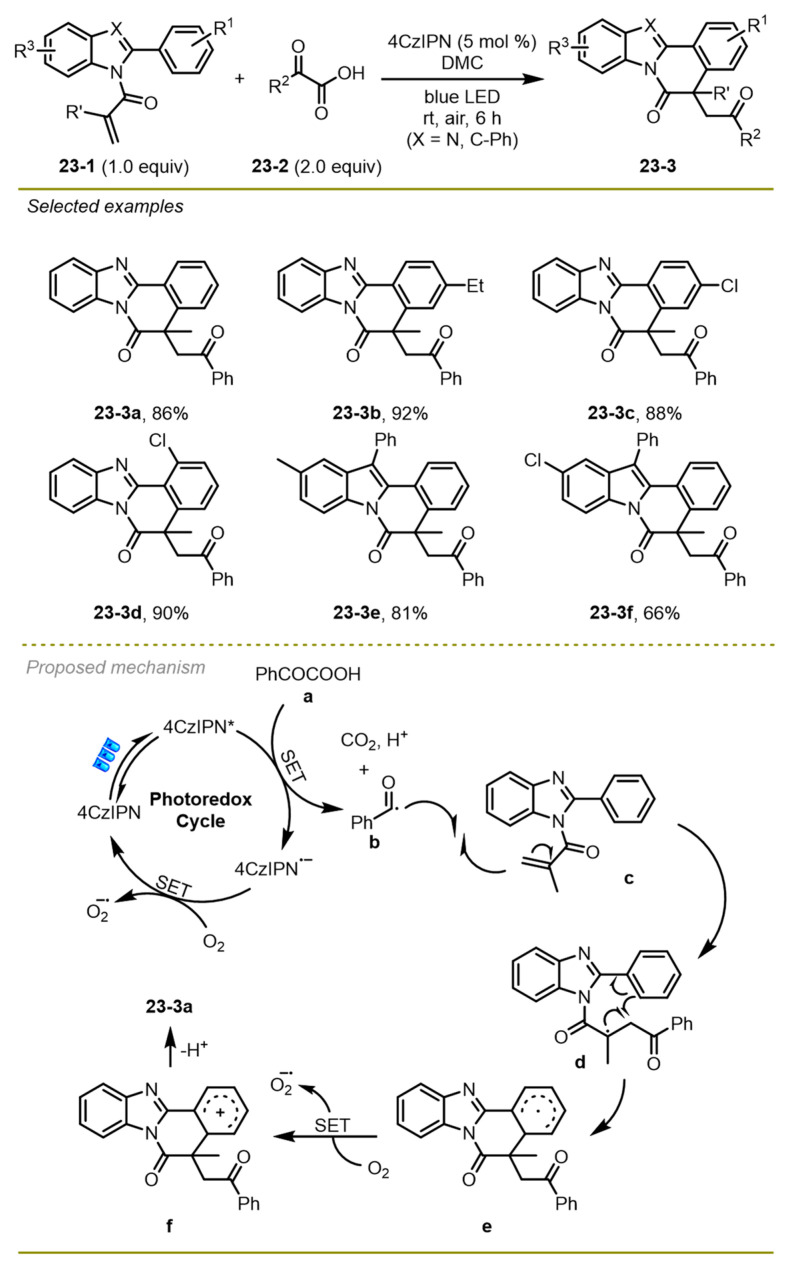
Metal-free visible-light photocatalytic decarboxylative arylation.

In 2021, a series of benzimidazo/indolo [2,1-a] isoquinolin-6(5H)-ones were prepared via metal-free visible-light photocatalytic decarboxylative arylation of readily available α-keto acids and 2-aryl indoles derivatives by Yu et al. (Scheme 23) [33]. This decarboxylative arylation strategy was well tolerated by aromatic α-keto acids and 2-aryl indoles with various electronic properties on the phenyl ring. Notably, the strategy employed 2,4,5,6-tetra(9H-carbazol-9-yl) isophthalonitrile (4CzIPN) as a photocatalyst in the mild conditions instead of expensive metal-based photocatalysts or stoichiometric amounts of hypervalent iodine reagents to greatly reduce the production cost.

A possible mechanism was proposed in Scheme 23. Initially, the visible-light-excited 4CzIPN* followed a single electron transfer with α-keto acids to afford benzoyl radical **b** and 4CzIPN^−^ along with the extrusion of CO_2_ and the release of H^+^. The O_2_ could oxidate 4CzIPN^−^ to form 4CzIPN and O_2_^−^, finishing the catalytic cycle. The addition of the benzoyl radical **b** followed intramolecular radical cyclization of the resulting stable tertiary radical **d**, generating aryl radical intermediate **e**. Subsequently, intermediate **e** was converted into cation **f** through a SET employing O_2_ as the oxidant and promoted a rapid deprotonation to produce the target products **23-3a**.

**Scheme 24:**
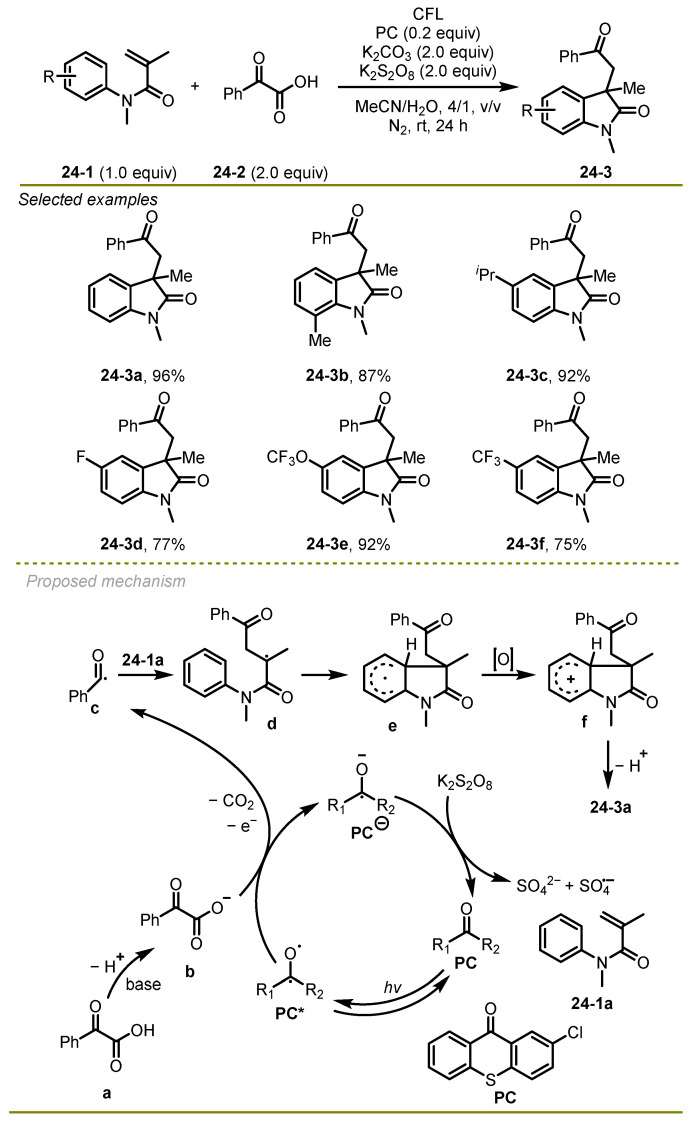
A metal-free visible-light photocatalytic cascade cyclization reaction.

A metal-free visible-light photocatalytic cascade cyclization reaction of α-keto acids and acrylamide derivatives was reported by Yu et al. in 2022, providing the valuable 2-oxindoles in moderate to good yields under mild conditions (Scheme 24) [34]. A variety of amides bearing either electron-deficient or electron-rich aryl substituents, such as Me, F, and OCF_3_, were proved to be valuable substrates, providing good yields of the products. The synthetic practicality of the protocol had been illustrated through gram-scale reaction and further transformations of the target oxindoles.

A hypothesized mechanism was proposed. Initially, α-keto acids followed base-mediated deprotonation and a single electron transfer with the excited state of photosensitizer 2-chlorothioxanthone (PC), forming the reductive anion of PC and benzoyl radical **c** with the release of CO_2_ gas. Subsequently, the addition of the benzoyl radical **c** onto the double bond of acrylamide derivatives **24-1a** followed intramolecular addition, producing intermediate **e**, which provided the desired product **24-3a** via oxidation and deprotonation reactions.

**Scheme 25:**
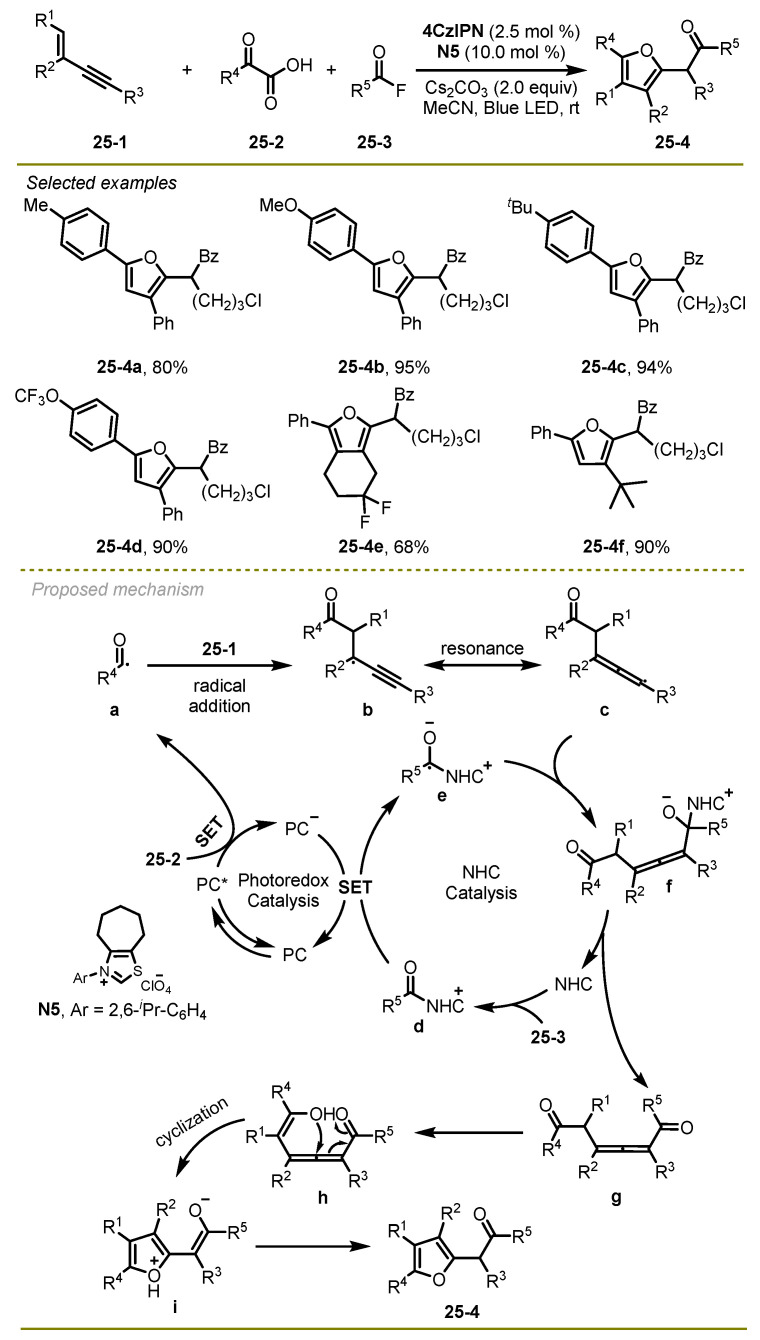
A visible light-induced NHC-catalyzed selective radical 1,4-diacyaltion/cycloisomerization cascade reaction.

In 2024, Luan et al. realized a visible light-induced NHC-catalyzed selective radical 1,4-diacyaltion/cycloisomerization cascade reaction of 1,3-enyne, α-keto acids and benzoyl fluoride, which could effectively construct a series of multisubstituted furan compounds (Scheme 25) [35]. This transformation would be an attractive approach for producing a heteroaromatic ring system featuring excellent functional group compatibilities and broad substrate scopes under mild conditions.

The proposed mechanism is shown in Scheme 25. First, a long-lived excited state 4CzIPN*, which was generated under blue LED irradiation, could undergo a reductive quenching process with α-keto acid and CO_2_-extrusion, producing acyl radical **a** and reducing state of 4CzIPN^•−^. Then, the former was added to the double bond of 1,3-enynes, leading to propargyl radical **b**, which is capable of reversibly converting to allenyl radical **c**. Simultaneously, benzoyl fluoride followed the acylation of NHC to produce intermediate **d**, followed a reduction reaction with the anion of 4CzIPN^•−^ to form the NHC-stabilized ketyl radical **e** and 4CzIPN. Subsequently, intermediate **e** could undergo cross coupling with radical **c** and dissociate from the NHC compound to produce the corresponding 1,6-diketone **g**, which could be converted into the desired product **25-4** via 5-exo-trig cyclization and isomerization.

**Scheme 26:**
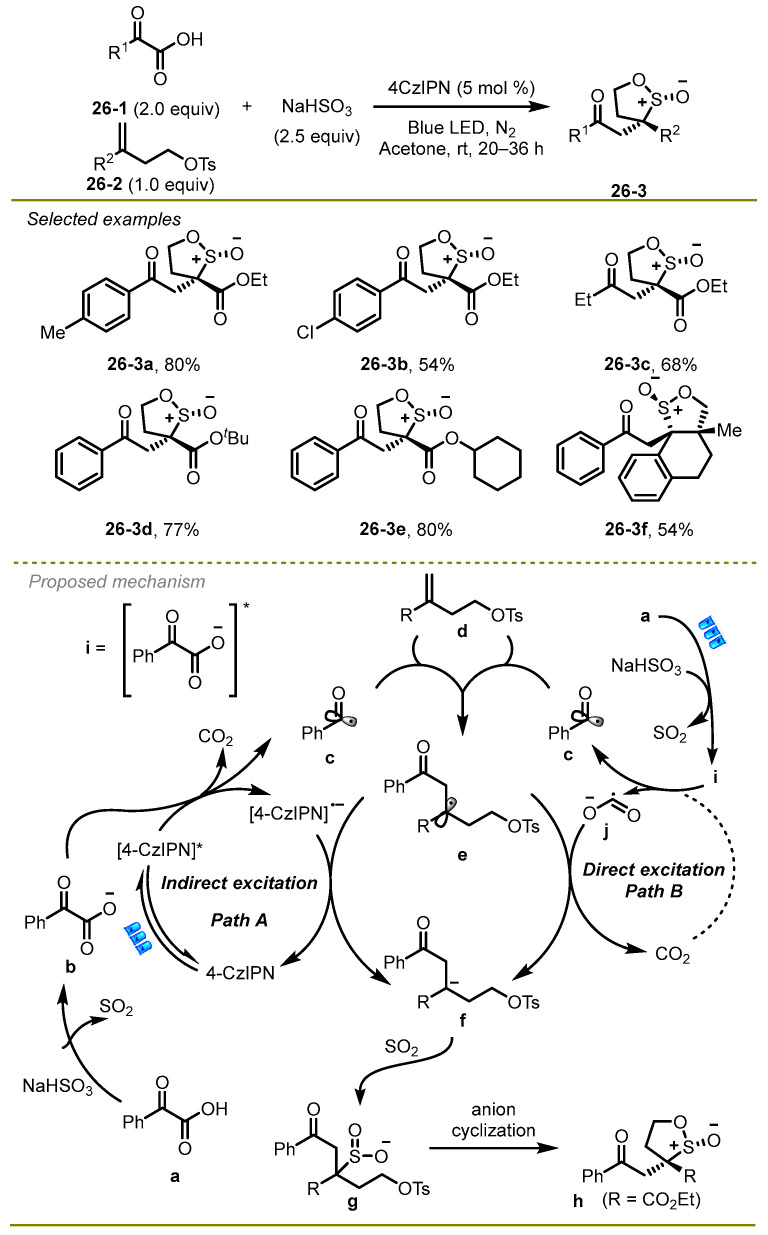
A further extension of base-free decarboxylative cross coupling of α-keto acids, alkenes, and sodium bisulfite.

In the same year, the Shu group discussed the further extension of base-free decarboxylative cross coupling of α-keto acids, alkenes, and sodium bisulfite via radical polar crossover cyclization (RPCC), which provided a facile and efficient protocol for the synthesis of β-keto sultine frameworks (Scheme 26) [36,37,38]. Notably, the economic and convenient sodium bisulfite served as both the SO_2_ source and the buffer agent in the protocol. A wide range of α-keto acids with different electronic effects, such as Me and Cl, were well suited to the reaction. The substituents on alkenes had no significant effect on producing the target products.

Based on a series of experimental outcomes, the possible mechanism was shown in schema 26. Initially, sodium bisulfite promoted the deprotonation of α-oxocarboxylic acid to obtain α-oxocarboxylate **b**, which could undergo SET with excited photocatalyst 4-CzIPN* to deliver an acyl radical **c** with the extrusion of CO_2_ (path A). Subsequently, the acyl radical **c** was added to alkene **d** to produce the radical **e**, followed by the reduction with 4-CzIPN^•−^ to deliver an anion **f**. Eventually, SO_2_ was inserted into anion **f** and produced a polar 5-exo-tet cyclization, providing the corresponding β-keto sultine **h**. It is worth noting that the acyl radical **c** could be generated without a photocatalyst via an energy excitation process upon irradiation with ultraviolet light (path B).

## 4. Conclusions

As highlighted in this review, the protocol for photoinduced decarboxylative acylation of α-keto acids has been consistently emerging as a highly appealing strategy for synthesizing valuable keto-containing acyclic and cyclic compounds. Particularly, α-keto acids have been shown to be an attractive alternative to moisture-sensitive acyl chlorides or toxic CO as an acylating agent, offering a straightforward and practical approach to a wide variety of useful ketones or related derivatives. Despite the previous positive aspects, as a rapidly growing field, there is still much more that needs to be explored in future research, such as (1) expanding the scope of acylation to include other unsaturated carbon-carbon bonds is highly desirable.; (2) how to control enantioselectivity in the decarboxylative acylation of α-keto acids using novel chiral catalysts or catalysis system; (3) how to develop more economic and environmentally friendly reactions as expensive transition metal photocatalysts are existed in some transformation; and (4) how to develop effective preparative methods to access α-keto acids. We aim to provide readers with a comprehensive understanding of the current state in this field and to make a contribution to future work.

## Data Availability

No data were created or analyzed in this study.
